# Supplementary material for: Conflict of interest in the peer review process: A survey of peer review reports
Source: PLoS One. 2023 Jun 8;18(6):e0286908. doi: 10.1371/journal.pone.0286908 (PMC10249818; doi:10.1371/journal.pone.0286908)

PLOS ONE

Conflict of Interest in the Peer Review Process: A Survey of Peer Review Reports

--Manuscript Draft--

**Manuscript Number:**	PONE-D-22-27587
**Article Type:**	Research Article
**Full Title:**	Conflict of Interest in the Peer Review Process: A Survey of Peer Review Reports
**Short Title:**	Conflict of Interest in the Peer Review Process: A Survey of Peer Review Reports
**Corresponding Author:**	Elie A. Akl<br>American University of Beirut<br>Beirut, LEBANON
**Keywords:**	Conflict of Interest; funding; authors; peer reviewers; editors; peer review process; editorial process
**Abstract:**	Objectives<br><br>To assess the extent to which peer reviewers and journals editors address study funding and authors' conflicts of interests (COI). Also, we aimed to assess the extent to which peer reviewers and journals editors reported and commented on their own or each other's COI.<br><br>Study design and methods<br><br>We conducted a systematic survey of original studies published in open access peer reviewed journals that publish their peer review reports. Using REDCap, we collected data in duplicate and independently from journals' websites and articles' peer review reports.<br><br>Results<br><br>We included a sample of original studies (N=144) and a second one of randomized clinical trials (N=115) RCTs.<br><br>In both samples, and for the majority of studies, reviewers reported absence of COI (70% and 66%), while substantive percentages of reviewers did not report on COI (28% and 30%) and only small percentages reported any COI (2% and 4%). For both samples, none of the editors whose names were publicly posted reported on COI. The percentages of peer reviewers commenting on the study funding, authors' COI, editors' COI, or their own COI ranged between 0 and 2% in either one of the two samples. 25% and 7% of editors respectively in the two samples commented on study funding, while none commented on authors' COI, peer reviewers' COI, or their own COI. The percentages of authors commenting in their response letters on the study funding, peer reviewers' COI, editors' COI, or their own COI ranged between 0 and 3% in either one of the two samples.<br><br>Conclusion<br><br>The percentages of peer reviewers and journals editors who addressed study funding and authors' COI and were extremely low. In addition, peer reviewers and journal editors rarely reported their own COI, or commented on their own or on each other's COI.
**Order of Authors:**	Adham Makarem, MD
	Rayan Mroué, BS
	Halima Makarem, MS, TD
	Laura Diab, BS
	Bashar Hassan, MD

Powered by Editorial Manager® and ProduXion Manager® from Aries Systems Corporation

	Joanne Khabsa, BSPharm, MPH
	Elie A. Akl, MD, MPH, PhD
**Additional Information:**	
**Question**	**Response**
**Financial Disclosure**<br><br>Enter a financial disclosure statement that describes the sources of funding for the work included in this submission. Review the submission guidelines for detailed requirements. View published research articles from *PLOS ONE* for specific examples.<br><br>This statement is required for submission and **will appear in the published article** if the submission is accepted. Please make sure it is accurate.<br><br>**Unfunded studies**<br>Enter: *The author(s) received no specific funding for this work.*<br><br>**Funded studies**<br>Enter a statement with the following details:<br>• Initials of the authors who received each award<br>• Grant numbers awarded to each author<br>• The full name of each funder<br>• URL of each funder website<br>• Did the sponsors or funders play any role in the study design, data collection and analysis, decision to publish, or preparation of the manuscript?<br>• **NO** - Include this sentence at the end of your statement: *The funders had no role in study design, data collection and analysis, decision to publish, or preparation of the manuscript.*<br>• **YES** - Specify the role(s) played.<br><br>* typeset	The author(s) received no specific funding for this work.
**Competing Interests**<br><br>Use the instructions below to enter a competing interest statement for this submission. On behalf of all authors, disclose any competing interests that could be perceived to bias this work—acknowledging all financial support and any other relevant financial or non-	The authors have declared that no competing interests exist.

financial competing interests.<br><br>This statement is required for submission and **will appear in the published article** if the submission is accepted. Please make sure it is accurate and that any funding sources listed in your Funding Information later in the submission form are also declared in your Financial Disclosure statement.<br><br>View published research articles from *PLOS ONE* for specific examples.<br><br>**NO authors have competing interests**<br><br>Enter: *The authors have declared that no competing interests exist.*<br><br>**Authors with competing interests**<br><br>Enter competing interest details beginning with this statement:<br><br>*I have read the journal's policy and the authors of this manuscript have the following competing interests: [insert competing interests here]*<br><br>* typeset	
**Ethics Statement**<br><br>Enter an ethics statement for this submission. This statement is required if the study involved:<br><br>• Human participants<br>• Human specimens or tissue<br>• Vertebrate animals or cephalopods<br>• Vertebrate embryos or tissues<br>• Field research<br><br>Write "N/A" if the submission does not require an ethics statement.<br><br>General guidance is provided below. Consult the submission guidelines for	N/A

detailed instructions. **Make sure that all information entered here is included in the Methods section of the manuscript.**<br><br>**Format for specific study types**<br><br>**Human Subject Research (involving human participants and/or tissue)**<br>• Give the name of the institutional review board or ethics committee that approved the study<br>• Include the approval number and/or a statement indicating approval of this research<br>• Indicate the form of consent obtained (written/oral) or the reason that consent was not obtained (e.g. the data were analyzed anonymously)<br><br>**Animal Research (involving vertebrate animals, embryos or tissues)**<br>• Provide the name of the Institutional Animal Care and Use Committee (IACUC) or other relevant ethics board that reviewed the study protocol, and indicate whether they approved this research or granted a formal waiver of ethical approval<br>• Include an approval number if one was obtained<br>• If the study involved *non-human primates*, add *additional details* about animal welfare and steps taken to ameliorate suffering<br>• If anesthesia, euthanasia, or any kind of animal sacrifice is part of the study, include briefly which substances and/or methods were applied<br><br>**Field Research**<br><br>Include the following details if this study involves the collection of plant, animal, or other materials from a natural setting:<br>• Field permit number<br>• Name of the institution or relevant body that granted permission	
**Data Availability**<br><br>Authors are required to make all data underlying the findings described fully available, without restriction, and from the time of publication. PLOS allows rare	Yes - all data are fully available without restriction

exceptions to address legal and ethical concerns. See the PLOS Data Policy and FAQ for detailed information.<br><br>A Data Availability Statement describing where the data can be found is required at submission. Your answers to this question constitute the Data Availability Statement and **will be published in the article**, if accepted.<br><br>**Important:** Stating 'data available on request from the author' is not sufficient. If your data are only available upon request, select 'No' for the first question and explain your exceptional situation in the text box.<br><br>Do the authors confirm that all data underlying the findings described in their manuscript are fully available without restriction?	
**Describe where the data may be found in full sentences. If you are copying our sample text, replace any instances of XXX with the appropriate details.**<br><br>• If the data are **held or will be held in a public repository**, include URLs, accession numbers or DOIs. If this information will only be available after acceptance, indicate this by ticking the box below. For example: *All XXX files are available from the XXX database (accession number(s) XXX, XXX.*).<br>• If the data are all contained **within the manuscript and/or Supporting Information files**, enter the following: *All relevant data are within the manuscript and its Supporting Information files.*<br>• If neither of these applies but you are able to provide **details of access elsewhere**, with or without limitations, please do so. For example:<br><br>*Data cannot be shared publicly because of [XXX]. Data are available from the XXX Institutional Data Access / Ethics Committee (contact via XXX) for researchers who meet the criteria for*	All relevant data are within the manuscript and its Supporting Information files.

access to confidential data.<br><br>*The data underlying the results presented in the study are available from (include the name of the third party and contact information or URL).*<br>• This text is appropriate if the data are owned by a third party and authors do not have permission to share the data.<br><br>* typeset	
Additional data availability information:	

Manuscript
Click here to access/download;Manuscript;MAKAREM Adham_COI manuscript.docx

Title:

Conflict of Interest in the Peer Review Process: A Survey of Peer Review Reports

Authors and Affiliations:

Author Name	Degree	Affiliation(s) (include city and country)	Email	Conflict of interest
Adham Makarem	MD	Faculty of Medicine, American University of Beirut, Beirut, Lebanon.	afm09@mail.aub.edu	None
Rayan Mroué	BS	Faculty of Medicine, American University of Beirut, Beirut, Lebanon.	ram83@mail.aub.edu	None
Halima Makarem	MS, TD	Faculty of Arts and Sciences, American University of Beirut, Beirut, Lebanon	hfm17@mail.aub.edu	None
Laura Diab	BS	Faculty of Medicine, American University of Beirut, Beirut, Lebanon.	lmd21@mail.aub.edu	None
Bashar Hassan	MD	Faculty of Medicine, American University of Beirut, Beirut, Lebanon.	bah24@mail.aub.edu	None
Joanne Khabsa	BSPharm, MPH	Clinical Research Institute, American	jk81@aub.edu.lb	Conducted studies on the topic of COI.

		University of Beirut, Beirut, Lebanon		
Elie A. Akl	MD, MPH, PhD	Department of Internal Medicine, American University of Beirut, Beirut, Lebanon; Department of Health Research Methods, Evidence, and Impact (HEI), McMaster University, Canada.	ea32@aub.edu.lb	Conducted studies on the topic of COI.

Corresponding author:

Elie A. Akl, MD, MPH, PhD

Department of Internal Medicine

American University of Beirut Medical Center

P.O. Box: 11-0236

Riad-El-Solh Beirut 1107 2020

Abstract:

Objectives: To assess the extent to which peer reviewers and journals editors address study funding and authors' conflicts of interests (COI). Also, we aimed to assess the extent to which peer reviewers and journals editors reported and commented on their own or each other's COI.

Study design and methods: We conducted a systematic survey of original studies published in open access peer reviewed journals that publish their peer review reports. Using REDCap, we collected data in duplicate and independently from journals' websites and articles' peer review reports.

Results: We included a sample of original studies (N=144) and a second one of randomized clinical trials (N=115) RCTs.

In both samples, and for the majority of studies, reviewers reported absence of COI (70% and 66%), while substantive percentages of reviewers did not report on COI (28% and 30%) and only small percentages reported any COI (2% and 4%). For both samples, none of the editors whose names were publicly posted reported on COI. The percentages of peer reviewers commenting on the study funding, authors' COI, editors' COI, or their own COI ranged between 0 and 2% in either one of the two samples. 25% and 7% of editors respectively in the two samples commented on study funding, while none commented on authors' COI, peer reviewers' COI, or their own COI. The percentages of authors commenting in their response letters on the study funding, peer reviewers' COI, editors' COI, or their own COI ranged between 0 and 3% in either one of the two samples.

Conclusion: The percentages of peer reviewers and journals editors who addressed study funding and authors' COI and were extremely low. In addition, peer reviewers and journal editors rarely reported their own COI, or commented on their own or on each other's COI.

Keywords: Conflict of Interest, funding, authors, peer reviewers, editors, peer review process, editorial process

Word count: 2613

Ethical approval: The study involves no human subjects and requires no ethical approval.

Competing interests: None.

What is new?

- This is one of the first studies that examines the extent to which peer reviewers and editors consider study funding and authors' COI, and the extent to which they report on their own COI.
- The percentages of peer reviewers and journals editors who addressed study funding & authors' COI were extremely low.
- Peer reviewers and journal editors rarely reported their own COI, or commented on their own or on each other's COI.

1. Introduction

The Institute of Medicine (IOM) defined conflict of interest (COI) as “circumstances that create a risk that professional judgments or actions regarding a primary interest will be unduly influenced by a secondary interest” [1, 2]. In the research field, conflict of interest arises when an individual prefers, or is perceived to prefer, their own interests which would increase the risk of biasing their judgments related to their professional obligations and responsibilities.

COI may involve a broad spectrum of interests. The financial interests are the most obvious. For example, a researcher may receive significant financial rewards from a pharmaceutical company with interest in the findings of their research. Such financial COIs are common among members of clinical guidelines panels [3, 4]. There is evidence that the quality of the research as well as guidelines may be negatively affected [5].

Non-financial interests can also affect the integrity of research. Such interests include career advancement, fame, social interests, and intellectual beliefs [6]. For instance, an editor may be conflicted when peer reviewing a colleague’s work. Intellectual COI is another type of non-financial COI discussed as far as two decades ago [7]. Lately, intellectual COI has been increasingly acknowledged [8] particularly in clinical practice guidelines (CPG) development [9, 10]. It has been defined as ‘‘academic activities that create the potential for an attachment to a specific point of view that could unduly affect an individual’s judgment about a specific recommendation’’ [11].

Researchers are expected to avoid and minimize COIs, and disclose them when they exist. The International Committee of Medical Journal Editors (ICMJE) has developed a specific and unified form for disclosure of COIs to facilitate and standardize authors' disclosures. Moreover, there are recommendations on how to manage and declare COIs among authors of clinical practice [12].

Peer reviewers and editors of journals play a key role in assessing and publishing research manuscripts [13]. On one hand, they need to assess the COI of authors and the funding source of the study. On the other hand, journal peer reviewers and editors may have their own conflict of interests that need to be disclosed [14].

In the current era, the standard for scientific publishing is to have research findings evaluated and published through a peer review process [15]. Peer-reviewed biomedical journals are publishing enormous number of articles each year. As of 2012, about 28000 scholarly journals published more than 2 million peer-reviewed articles [16]. An optimal scientific peer review process is essential to maintain the integrity of the scientific research and to support evidence-based practice.

There is increased media attention to the reported conflicts and concerns about the impact of industry-sponsorship [17, 18]. A transparent handling of conflicts of interest is essential for the public trust in the scientific process and the credibility of peer-reviewed published articles [6]. The reporting of authors' disclosure of conflicts of interest in publications has become the standard [14]. In order to facilitate and standardize the process of authors' disclosures, the

International Council of Medical Journal Editors (ICMJE) has developed a form for the disclosure of COI [6]. The authors are required to declare via this form all financial and non-financial benefits or personal relationships that might bias their work.

COI issues are relevant to all participants in the peer-reviewed publication process – including peer reviewers, editors, and the editorial board members of the journals. The peer reviewers' role is to critically assess the manuscript, by constructively commenting on the scientific work, and suggesting how to improve it [19]. Moreover, peer-reviewers and editors are expected to reflect and comment on the authors' disclosures of conflicts of interest. There are questions about the effectiveness of the current system of COI disclosures. A randomized controlled study found that providing journal reviewers with authors' conflict of interest information had no significant effect on their rating of the quality of the manuscript [20].

Similarly, journal editors have a core role in managing the review process, through assessing the peer reviewers' reports and making the final decision on acceptance for publication. In addition, editors can significantly impact the integrity, quality and fairness of the peer review process by how they select the peer reviewers and managing any misconducts by authors or reviewers [21]. Editors are also expected to review and consider the authors' disclosures of conflict of interest as part of the peer review process. However, this aspect of their role has not been studied yet.

According to the ICMJE, peer reviewers and editors have to disclose their own conflicts of interest [6]. In some cases, those invited to peer review and editors may need to rescue

themselves from being involved. Little is known about the practices and policies of journals regarding disclosures of conflict of interest among peer-reviewers and editors to public [14]. A study assessing the COI policies of health policy and services journals, found only one that described how the COIs of the editorial team are managed during the editorial process [22].

The objective of this study was to assess the extent to which peer reviewers and journal editors address authors' conflicts of interests and study funding. Also, we aimed to assess the extent to which peer reviewers and journals editors reported and commented on their own or each other's COI.

2. Methods:

We included two samples in this study. First, we included a sample with any type of original research. However, we found that a very low percentage (9%) had at least one author reporting presence of COI. Given this would not allow us achieve the study objective, we decided to collect a second sample restricted to randomized controlled trials (RCTs) as a survey had found that more than half of clinical trials had authors reporting presence of COI [23].

2.1. Eligibility criteria

We included journals that publish in the health field and in English, are indexed in Medline, and publish all their peer review reports. We included only original articles that are peer reviewed.

2.2. *Selecting reports*

We used the Directory of Open Access Journals (DOAJ) as an initial list of journals. Then, we filtered the journals by language (English) and subject (Medicine). Then, we selected our samples according to the eligibility criteria. For our first sample, we included the latest two original publications from each journal that had peer review reports. For our second sample, we included the latest two randomized controlled trials (RCTs) that had peer review reports. Teams of two reviewers implemented the selection process in duplicate and independently. They resolved any disagreements through discussion, or with the help of a third reviewer if needed.

2.3. *Data abstraction*

We developed data extraction form using the Research Electronic Data Capture (REDCap) tool hosted at the American University of Beirut. We also developed detailed instructions. After conducting calibration exercises, teams of two reviewers abstracted data in duplicate and independently. They resolved any disagreements through discussion, or with the help of a third reviewer if needed.

We abstracted the following data:

- Characteristics of the journal (including impact factor, field)
- Characteristics of the publication (primary research vs. systematic review; source of funding)
- Whether the authors reported their COI and the study funding source

- Whether the peer reviewers commented on authors' COI and the study funding source
- Whether the journal editors commented on authors' COI and the study funding source
- Whether the peer reviewers reported their own COI
- Whether the journal editors reported their own COI
- Whether authors and editors commented on the peer reviewers' COI
- Whether authors and peer reviewers commented on the editors' COI

2.4. Data analysis

We exported all data from REDCap to an Excel sheet for data cleaning and consistency checks. We conducted quantitative descriptive analyses of all variables. We used percentage for categorical variables and median and interquartile range for continuous variables. We also conducted a thematic analysis of comments extracted.

3. Results:

3.1. Search results

Figure 1 shows the selection process for the 2 samples included in this study. From the initial list of 150 open peer reviewed journals identified from the DOAJ, we included 72 journals meeting our eligibility criteria.

For our first sample, we included 144 articles, two from each of the 72 eligible journals. For our second sample, 58 journals were eligible and published randomized controlled trials. One of these journals published only one RCT. Consequently, we included a total of 115 articles.

Figure 1: The selection process for the 2 samples included in this study.

3.2. General characteristics

Table 1 presents the general characteristics of the journals included in the two samples. For both samples, the majority of journal were from the clinical field (80% and 83%), were published in BioMed Central (BMC) (85% and 89%), and were indexed in Medline (97% and 100%). For the majority of journals peer review was by invitation (99% and 98%), was conducted pre-publication (90% and 91%), and did not reveal the identity of the peer reviewers (56% and 64%).

Table 2 presents the general characteristics of publications included in the two samples. The percentages of RCTs were 3% for sample 1 and 100% for sample 2. For both samples small percentages had COVID-19 as topic (8% and 3%), the median number of authors was 6, the majority had their first author primarily affiliated with academia (87% and 93%), and the majority had the first author affiliated with a high-income country (63% and 61%).

Table 3 presents characteristics of the peer review process. For both samples, the median for number of rounds of peer review, and the number of rounds of revision, and the number of peer reviewers was 2. In the two samples, the median of the number of editors involved was 1. Only 11% and 13% of editors' letters to the authors were posted publicly, and of those 63% and 53% respectively provided the name of the editor.

3.3. *Funding of the study*

Table 4 presents the characteristics of funding of the included studies. For both samples, the majority of studies were reported as funded (67%and 90%) and included a statement on the funder's role (57% and 59%). The top two sources of funding were governmental (61% and 46%) and internal funding (41% and 46%).

3.4. *Declaration of conflict of interest by authors, peer reviewers, and editors*

Table 5 summarizes the declaration of conflict of interest by authors, peer reviewers, and editors. For both samples, the majority of studies had all authors report the absence of COI (89% and 68%). For both samples, the majority of studies had reviewers identified by name (55% and 56%), reported absence of COI (70% and 66%). Substantive percentages did not report on COI of reviewers (28% and 30%) while only small percentages reported any COI (2% and 4%). For both samples, none of the editors whose names were publicly posted reported on COI.

3.5. *Commenting on declarations of conflict of interest*

Table 6 summarizes the extent of authors, peer reviewers and editors commenting on study funding and conflicts of Interest. The percentages of peer reviewers commenting on the study funding, authors' COI, editors' COI, or their own COI ranged between 0 and 2% in either one of the two samples. 25% and 7% of editors respectively in the two samples commented on study funding, while none commented on authors' COI, peer reviewers' COI, or their own COI. The percentages of authors commenting in their response letters on the study funding, peer

reviewers' COI, editors' COI, or their own COI ranged between 0 and 3% in either one of the two samples. Figure 2 below illustrates these results among our 2 samples.

Figure 2: Commenting on own and other's COI by authors, peer reviewers, and editors

4. Discussion:

4.1.Summary of findings

In summary, the percentages of peer reviewers and editors commenting on study funding and authors' COI were extremely low. In addition, peer reviewers and journal editors rarely reported their own COI, or commented on their own COI, or on each other's COI.

4.2.Strengths and limitations

To our knowledge, this is one of the first studies that examines the extent to which peer reviewers and editors consider study funding and authors' COI, and the extent to which they report on their own COI. To ensure our findings are informative, we went beyond our original plan and included a second sample that involved a higher percentage of articles whose authors reported presence of COI, compared with our first sample. We found consistent results for the two samples. One limitation of this study is that our samples represent the peer review processes of articles that ended up being accepted for publication, but not of those that were not published or those that were published in non-open peer review journal.

4.3.Comparison to similar studies

The percentage of articles with at least one author reporting presence of COI for our first sample was only 9%. This percentage is much lower compared to that of systematic reviews (41%) and clinical trials (57%) [23, 24]. For our second sample, the percentage was higher at 30% because we included only randomized clinical trials. In addition, our findings that peer reviewers and editors are not reporting their own COI to a good extent are consistent with other studies in the literature. A recent study highlighted the lack of discussion surrounding editorial conflicts of interest in public health journals [25]. The authors noted that the editorial COI policies did not adhere to guidelines and that these policies need improvements [25].

4.4.Implications for practice and research

The results of our project provide insight about the COI disclosure of peer reviewers, authors and editors. This may help journals to develop policies to improve how COI is declared and managed during their editorial processes. It would be relevant to conduct qualitative research to explore why some peer reviewers and editors are commenting on authors' COI and on the study funding and others are not. Such study may help in developing strategies to improve COI declaration and management during the peer review process.

Tables:

Table 1: General characteristics of included journals

	Sample 1 (N=72)[1]	Sample 2 (N=58)[1]
	n (%)	n (%)
Impact factor	Med= 2.674<br>IQR = [2.28 – 3.27]	Med= 2.652<br>IQR = [2.28 – 3.26]
Field*		
Basic sciences	34 (47)	29 (50)
Clinical	57 (80)	48 (83)
Public Health	41 (57)	31 (53)
Health systems and policy	23 (32)	17 (29)
Other	4 (5)[2]	8 (14)[3]
Publisher		
Taylor & Francis	4 (6)	4 (7)
BioMed Central (BMC)	61 (85)	52 (89)
Other	7 (9)[4]	2 (4)[5]
Journal indexed in Medline	70 (97)	58 (100)
Peer review only by invitation process		
No (open participation)	0 (0)	0 (0)
Yes (invitation needed)	71 (99)	57 (98)
Not reported	1 (1)	1 (2)
Peer review report:		
Pre-publication review	65 (90)	53 (91)
Post-publication review	7 (10)	5 (9)

[1] sample 1 includes articles of any type of original research. Sample 2 is restricted to only randomized controlled trials (RCTs)
[2] n=2: Computational and Biotechnologies; n=1: Biomedical; n=1: Translational
[3] n=3: Research Methodologies; n=2: monitoring and computational modeling; n=1: Health information technology; n=1: Health financing and Economics; n=1: Medical Education and Training
[4] n=1: Springer; n=1: Oxford University Press; n=1: Cambridge University Press; n=1: BMJ Publishing Group; n=1: Association for Medical Education in Europe (AMEE); n=1: Wellcome; n=1:Physiopedia
[5] n=1: Springer; n=1: Wellcome

Open identity of peer reviewer	32 (44)	21 (36)

*Total does not add up to 100 due to overlap

Table 2: General characteristics of the included publications

	Sample 1 (N=144)[6]	Sample 2 (N=115)[6]
	n (%)	n (%)
Type of research:		
Randomized controlled trials (RCTs)	5 (3)	115 (100)
Non-randomized studies	64 (45)	0 (0)
qualitative	18 (13)	0 (0)
survey	5 (3)	0 (0)
systematic reviews	13 (9)	0 (0)
case reports	17 (12)	0 (0)
Other:	22 (15)	0 (0)
COVID 19 topic	11 (8)	2 (3)
Number of authors	Med = 6, IQR = [4 – 8]	Med = 6, IQR = [5 – 10]
First author's primary affiliation:		
Academic	125 (87)	107 (93)
Governmental	8 (6)	4 (3)
Intergovernmental	0 (0)	0 (0)
Not for profit organization, other than academic	6 (4)	3 (3)
Private for profit	5 (3)	1 (1)
Other	0 (0)	0 (0)

[6] sample 1 includes articles of any type of original research. Sample 2 is restricted to only randomized controlled trials (RCTs)

Classification of the affiliated country of the first author:		
High income	90 (63)	70 (61)
Upper-middle income	33 (23)	17 (15)
Lower-middle income	15 (10)	23 (20)
Low income	6 (4)	5 (4)

Table 3: Characteristics of the peer review process

	**Sample 1 (N=144)** [7]	**Sample 2 (N=115)** [7]
Number of rounds of peer review	Med = 2, IQR = [1 – 2]	Med = 2, IQR = [1 -- 2]
Number of rounds of revision	Med = 2, IQR = [2 – 4]	Med = 2, IQR = [2 – 3]
Number of peer reviewers, per study	Med = 2, IQR = [2 – 2.25]	Med = 2, IQR = [2 – 2]
Number of reviewers, total	330	263
Peer reviewers identified by name	180 (55) [8]	146 (56) [8]
Number of editors involved, per study	Med = 1, IQR = [1 – 1]	Med = 1, IQR = [1 – 1]
Public posting of editors' letter to the authors	16 (11) [9]	15 (13) [9]
Editor identified by name	10 (63) [10]	8 (53) [10]

[7] sample 1 includes articles of any type of original research. Sample 2 is restricted to only randomized controlled trials (RCTs)
[8] denominator is the total number of reviewers: /330 in sample 1 and /263 in sample 2
[9] denominator is the number of editors involved: /144 in sample 1 and /115 in sample 2
[10] denominator is the number of editors who publicly posted letters to the authors: /16 in sample 1 and /15 in sample 2

Table 4: Funding of the included studies

	Sample 1 (N=144) [11]	Sample 2 (N=115) [11]
	n (%)	n (%)
Funding status		
Not funded	38 (26)	9 (8)
Funded	97 (67)	103 (90)
Not reported	9 (7)	3 (2)
Reported Source(s) of Funding	N=97 *	N=103 *
Internally funded	40 (41)	47 (46)
Governmental	59 (61)	47 (46)
Private for Profit	11 (11)	16 (16)
Private not for Profit	19 (20)	22 (21)
Intergovernmental	12 (12)	8 (8)
Academic	14 (14)	8 (8)
Statement of the funder's role	55/97 (57)	61/103 (59)

*Total does not add up to 100 due to overlap

[11] sample 1 includes articles of any type of original research. Sample 2 is restricted to only randomized controlled trials (RCTs)

Table 5: Declaration of conflict of interest of authors, peer reviewers, and editors

	**Sample 1** [12]	**Sample 2** [12]
Authors reporting of COI	N=144	N=115
Reports absence of COI	129 (89)	79 (68)
Reports presence of COI (at least 1)	13 (9)	34 (30)
Does not report on COI	2 (2)	2 (2)
Peer reviewers' reporting of COI	N=330	N=263
Reports absence of COI	232 (70)	175 (66)
Reports COI	7 (2)	9 (4)
Does not report on COI	91 (28)	79 (30)
Editor's reporting of COI	N=16	N=15
Reports COI	0 (0)	0 (0)
Reports absence of COI	0 (0)	0 (0)
Does not report on COI	16 (100)	15 (100)

[12] sample 1 includes articles of any type of original research. Sample 2 is restricted to only randomized controlled trials (RCTs)

Table 6: Commenting on study funding and conflicts of Interest by authors, peer reviewers and editors

	**Sample 1** [13]	**Sample 2** [13]
Authors (comments in their response letters)	N=144	N=115
Authors commenting on study funding	5 (3)	1 (1)
Authors commenting on peer reviewers' COI	0 (0)	0 (0)
Authors commenting on the editors' COI	0 (0)	0 (0)
Authors commenting on their own COI	2 (2)	0 (0)
Peer reviewers	N=330	N=263
Peer reviewer commenting on study funding	1 (1)	4 (2)
Peer reviewer commenting on authors' COI	3 (1)	2 (1)
Peer reviewer commenting on their own COI	2 (1)	1 (1)
Peer reviewer commenting on editors' COI	0 (0)	0 (0)
Editors	N=16	N=15
Editor commenting on study funding	4 (25)	1 (7)
Editor commenting on author's COI	0 (0)	0 (0)
Editor commenting on their own COI	0 (0)	0 (0)
Editor commenting on peer reviewers' COI	0 (0)	0 (0)

[13] sample 1 includes articles of any type of original research. Sample 2 is restricted to only randomized controlled trials (RCTs)

References:

1. Greenfield, S., et al., *Clinical practice guidelines we can trust. Washington, DC: Institute of Medicine; 2011*. 2017.
2. Field, M.J. and B. Lo, *Conflict of interest in medical research, education, and practice.* 2009.
3. Mendelson, T.B., et al., *Conflicts of interest in cardiovascular clinical practice guidelines.* Arch Intern Med, 2011. **171**(6): p. 577-84.
4. Neuman, J., et al., *Prevalence of financial conflicts of interest among panel members producing clinical practice guidelines in Canada and United States: cross sectional study.* BMJ, 2011. **343**: p. d5621.
5. Cosgrove, L., et al., *Conflicts of interest and the quality of recommendations in clinical guidelines.* J Eval Clin Pract, 2013. **19**(4): p. 674-81.
6. Editors, I.C.o.M.J., *Recommendations for the conduct, reporting, editing, and publication of scholarly work in medical journals*. 2018.
7. Marshall, E., *When does intellectual passion become conflict of interest?* Science, 1992. **257**(5070): p. 620-3.
8. Levinsky, N.G., *Nonfinancial conflicts of interest in research.* N Engl J Med, 2002. **347**(10): p. 759-61.
9. Lederer, E.D., *Development of clinical practice guidelines: are we defining the issues too narrowly?* Clin J Am Soc Nephrol, 2007. **2**(2): p. 207.
10. Detsky, A.S., *Sources of bias for authors of clinical practice guidelines.* CMAJ, 2006. **175**(9): p. 1033, 1035.
11. Guyatt, G., et al., *The vexing problem of guidelines and conflict of interest: a potential solution.* Ann Intern Med, 2010. **152**(11): p. 738-41.
12. Lo, B. and M.J. Field, *Institute of Medicine (US) Committee on Conflict of Interest in Medical Research, Education, and Practice, eds.(2009): Conflict of Interest in Medical Research, Education, and Practice*. Washington (DC): National Academies Press (US). www. ncbi. nlm. nih. gov ….
13. Glonti, K., et al., *A scoping review on the roles and tasks of peer reviewers in the manuscript review process in biomedical journals.* BMC Med, 2019. **17**(1): p. 118.
14. Cooper, R.J., et al., *Conflict of Interest Disclosure Policies and Practices in Peer-reviewed Biomedical Journals.* J Gen Intern Med, 2006. **21**(12): p. 1248-52.
15. Davis, C.H., et al., *Reviewing the review: a qualitative assessment of the peer review process in surgical journals.* Res Integr Peer Rev, 2018. **3**: p. 4.
16. Manchikanti, L., et al., *Medical journal peer review: Process and bias.* Pain Physician, 2015. **18**(1): p. E1.
17. Davidoff, F., et al., *[Sponsorship, authorship and accountability].* Tidsskr Nor Laegeforen, 2001. **121**(21): p. 2531-2.
18. Fontanarosa, P.B., A. Flanagin, and C.D. DeAngelis, *Reporting conflicts of interest, financial aspects of research, and role of sponsors in funded studies.* JAMA, 2005. **294**(1): p. 110-1.

19. Evans, A.T., et al., *The characteristics of peer reviewers who produce good-quality reviews.* Journal of general internal medicine, 1993. **8**(8): p. 422-428.
20. John, L.K., et al., *Effect of revealing authors' conflicts of interests in peer review: randomized controlled trial.* bmj, 2019. **367**: p. l5896.
21. Resnik, D.B. and S.A. Elmore, *Ensuring the quality, fairness, and integrity of journal peer review: A possible role of editors.* Science and Engineering Ethics, 2016. **22**(1): p. 169-188.
22. Khamis, A.M., et al., *Requirements of health policy and services journals for authors to disclose financial and non-financial conflicts of interest: a cross-sectional study.* Health Res Policy Syst, 2017. **15**(1): p. 80.
23. Hakoum, M.B., et al., *Authors of clinical trials reported individual and financial conflicts of interest more frequently than institutional and nonfinancial ones: a methodological survey.* Journal of clinical epidemiology, 2017. **87**: p. 78-86.
24. Hakoum, M.B., et al., *Reporting of financial and non-financial conflicts of interest by authors of systematic reviews: a methodological survey.* BMJ open, 2016. **6**(8): p. e011997.
25. Ralph, A., M. Petticrew, and A. Hutchings, *Editor and peer reviewer financial conflict of interest policies in public health journals.* European Journal of Public Health, 2020. **30**(6): p. 1230-1232.

Figure 1: The selection process for the 2 samples included in this study.

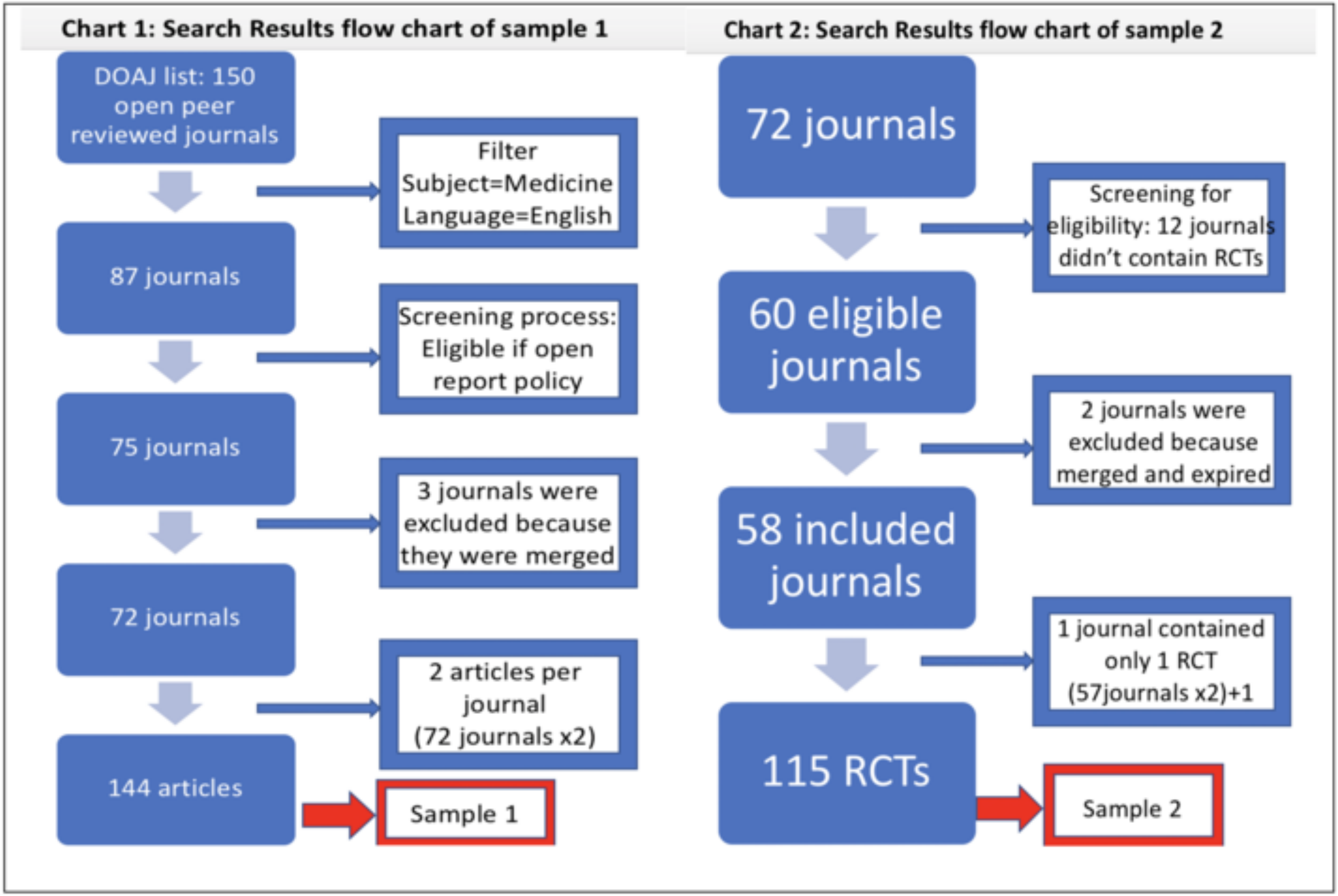

Figure 2: Commenting on own and other's COI by authors, peer reviewers and editors

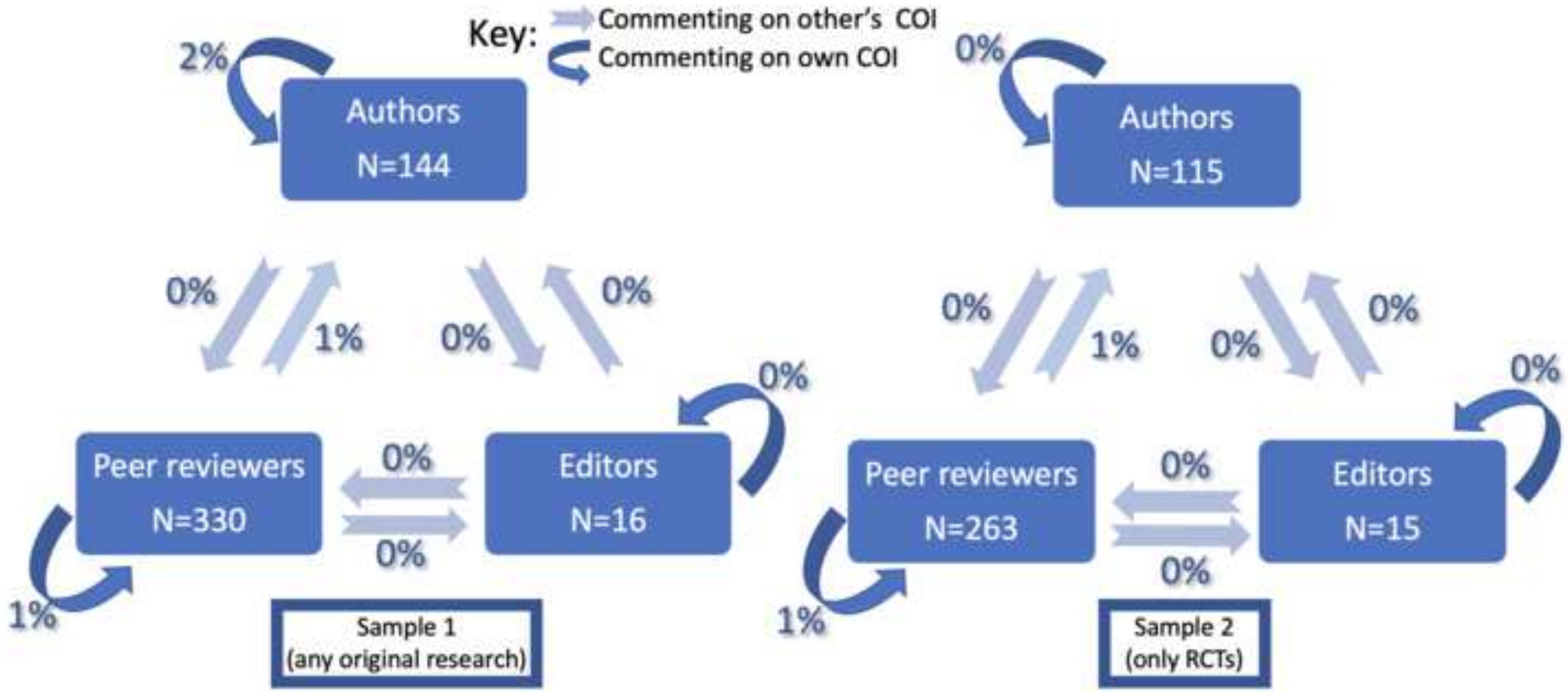

 Click here to access/download;Figure;Graphical Abstract_COI manuscript.docx

Re: "Conflict of Interest in the Peer Review Process: A Survey of Peer Review Reports"

Graphical Abstract:

Figure 1: Commenting on own and other's COI by authors, peer reviewers and editors

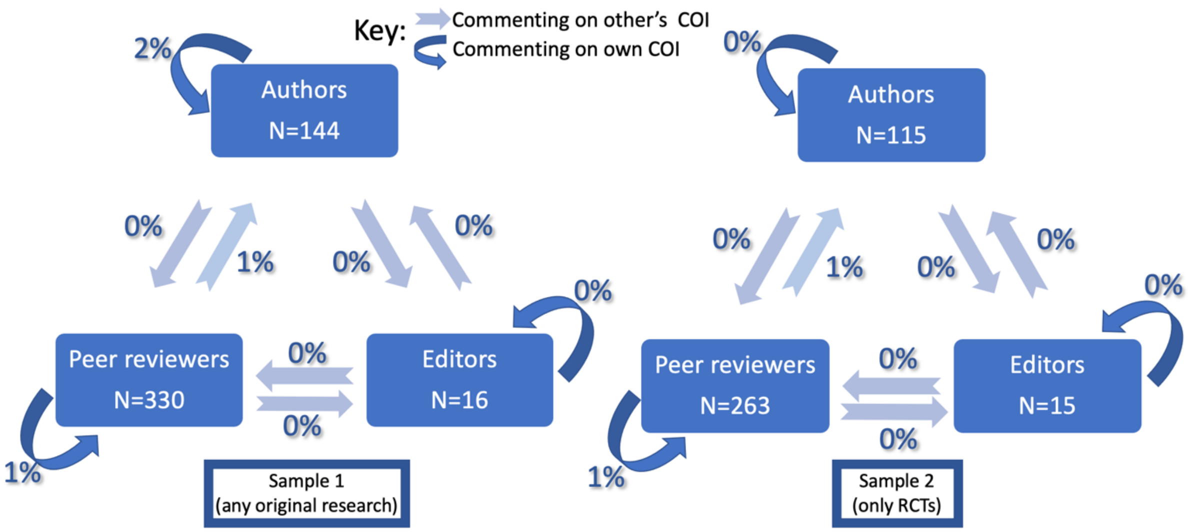

Supplement: Attachment — Submitted filename: PONE-D-22-27587_reviewer_Ss Comments_2Feb23.pdf [file pone.0286908.s002.pdf]
