# Supplementary material for: Conflict of interest in the peer review process: A survey of peer review reports
Source: PLoS One. 2023 Jun 8;18(6):e0286908. doi: 10.1371/journal.pone.0286908 (PMC10249818; doi:10.1371/journal.pone.0286908)
Supplement: Attachment — Submitted filename: Response to Reviewers.docx [file pone.0286908.s003.docx]

**Department of Internal Medicine**

**American University of Beirut**

April 2^nd^, 2023

Dr. Fares Alahdab

Academic Editor, PLOS ONE journal

Re: “Conflict of Interest in the Peer Review Process: A Survey of Peer Review Reports”

Dear Dr. Alahdab,

Thank you for the opportunity to revise and resubmit our above titled manuscript to the PLOS ONE Journal. We thank you and the reviewers for taking the time to review the manuscript. We found the comments and suggestions very constructive and used them to improve our manuscript.

Kindly find below a point-by-point response to the reviewers’ comments and a description of the changes made to the manuscript.

We look forward to the outcome of the editorial review of our revised manuscript.

Sincerely,

Elie A. Akl, MD, MPH, PhD

Department of Internal Medicine

American University of Beirut

P.O. Box: 11-0236

Riad-El-Solh Beirut 1107 2020

Beirut – Lebanon

Phone: 00961 1 350 000

[ea32@aub.edu.lb](about:blank)

**The reviewers’ comments are listed below in regular font. Our responses to these comments are in bold. Extracts from the text are in italic font. We have indicated the sections where revisions have been made in our manuscript.**

**Reviewer #1:**

The very subject of this paper challenges the peer reviewer to be scrupulous and meticulous in their review. The PLOS peer review process asks me to justify my responses to 4 specific questions.

1. I find the survey approach taken, and the adjustments made, to be appropriate to the question. The authors discuss the limitations of their sample but this is a consequence of issues beyond their control. The conclusions reached are appropriate.

2. The statistical analysis is straightforward, appears to be correctly undertaken and is informative.

3. The authors declare that they have made all the underlying data available, much of it is in the manuscript.

4. The manuscript is clear and easy to follow and written in standard English.

**Response: Thank you for positive assessment on each of the four questions.**

Additional comments

This paper is a salutary reminder of the full extent of our responsibilities as researchers, reviewers and publishers. The research community has come along way in requiring declarations of conflicts of interests from primary researchers, but we clearly have some way to go with how we review this information – saying we have made a declaration should not be enough in itself – but this paper shows us that for the majority of peer reviewers and editors it is – there is not enough checking and alerting the readers of COI going on.

I thought the authors did a good job of presenting their findings in a factual, non-judgmental fashion – they could have said more about the potential implications, but I think they have made the right choice, it is not difficult for the reader to see where these findings lead.

**Response: Thank you again for the summary and very positive feedback.**

There were some interesting findings that it would have been interesting to hear more about, for example, more on "intellectual COI" and how routinely that is declared.

**Response: Thank you for the suggestion. We went back and categorized the declarations of interests according to a framework that we recently published and that we now also refer to in the introduction and methods section. As a results, here is what we found in terms of categories of interests declared (Refer to footnotes in table 5):**

***“4/7 had Individual financial interests with direct financial benefit, 2/7 had Individual personal interests, 1/7 had Individual intellectual interests.***

***6/9 had Individual financial interests with direct financial benefit, 3/9 had Individual personal interests.”***

Another interesting snippet was that the majority of authors in the papers sampled came from high income countries. Perhaps the acknowledged gap in analysis of papers rejected would tell us more.

**Response: Yes, the distribution of the affiliated country of first authors of our sample were around 60% high income countries, 35% upper-middle and lower-middle income, and the rest 5% were low-income countries. We do address that in the ‘limitations’ section of the discussion:**

***“One limitation of this study is that our samples represent the peer review processes of articles that ended up being accepted for publication, but not of those that survive the peer review process or those that were published in non-open peer review journal.”***

**We also added the following to the ‘implications for research’ section of the discussion:**

***“Also, it would be important to conduct a similar study of articles that underwent the peer review process but did not survive the peer review process or of articles that were published in non-open peer review journal.”***

Finally, across all samples, only one editor used in the peer review process and no COI declared - there are some real lessons for us all in this paper.

**Response: We agree, this was an interesting and alarming finding.**

**Reviewer #2:**

This is a very important study.

**Response: Thank you for your comment**

Please add an explanation before publication.

Please explain a little bit more about Research Electric Data Capture (REDCap)? I do not understand your description " We developed data extraction form using the Research Electronic Data Capture (REDCap) tool hosted at the American University of Beirut. We also developed detailed instructions. After conducting calibration exercises, teams of two reviewers abstracted data in duplicate and independently. They resolved any disagreements through discussion, or with the help of a third reviewer if needed."

1) Would you explain the process in detail? Is REDCap validated?

**Response: Thank you for raising this question. REDCap (Research Electronic Data Capture) is a web-based software platform designed for clinical research studies and surveys and is widely used. According to their website is has been used by 6452 research institutions in 151 countries for 1.7 million project leading to 23.8 thousand articles (see here: https://www.project-redcap.org). REDCap provides researchers with a secure and customizable platform for capturing, managing, and analyzing research data. It provides features for creating surveys, managing study participants, tracking study progress, and exporting data in a variety of formats for statistical analysis. It is validated and has features for ensuring data security and compliance with privacy regulations. REDCap is available to researchers at no cost and is supported by a large community of users and developers. We have added the following text along with a citation:**

***We collected and managed study data using the Research Electronic Data Capture (REDCap) tool hosted at the American University of Beirut (25). REDCap is a secure, web-based software platform designed to support data capture for research studies.***

2) Also, the readers cannot access the system and check the data reproducibility?

**Response: In terms of eligible journals, the readers can visit the** [**DOAJ website**](https://doaj.org/)**, apply** [**our filters**](https://doaj.org/search/journals?source=%7B%22query%22%3A%7B%22bool%22%3A%7B%22must%22%3A%5B%7B%22terms%22%3A%7B%22index.schema_codes_tree.exact%22%3A%5B%22LCC%3AR%22%5D%7D%7D%2C%7B%22terms%22%3A%7B%22index.language.exact%22%3A%5B%22English%22%5D%7D%7D%2C%7B%22terms%22%3A%7B%22bibjson.editorial.review_process.exact%22%3A%5B%22Open%20peer%20review%22%5D%7D%7D%5D%7D%7D%2C%22size%22%3A50%2C%22sort%22%3A%5B%7B%22created_date%22%3A%7B%22order%22%3A%22desc%22%7D%7D%5D%2C%22track_total_hits%22%3Atrue%7D)**, and reach our selected sample of journals. We have also included as an appendix all raw extracted data to allow others to assess reproducibility. Here is what we say in the ‘data analysis’ section:**

***“We are sharing that Excel sheet to make all raw data available (see Appendix 1).”***

**Reviewer #3:**

The manuscript is a technically sound research and the presented data support the conclusions reached. The sampling and sample size are adequate, statistical analyses done are simple but adequately serves the purpose of the research. All the data in the result section were adequately presented. The language of the manuscript is simple and clear, with no grammatical error noted.

**Response: Thank you for the very positive feedback.**

**Reviewer #4:**

1. An important theme. Will indeed be relevant towards improving reporting of COI. I have a number of comments seeking clarification and making statements clearer. Please see my comments embedded in the manuscript.

**Response: Thank you for your constructive feedback. Please see below how we addressed each of your comments seeking clarification:**

- Methods section:

A. Original research relating to health? More specifics will be helpful.

If it is only biomedical research, it still requires to be defined for readers to know the scope of studies included in the study.

Later it says 'medicine'.

As mentioned earlier, 'health' would cover a much larger canvas compared to 'medicine'.

Suggest to clarify and if needed to use one single terms - health or medicine.

**Response: Thank you for pointing this out. We clarified that the study addresses medical research as determined by our eligibility criteria and journal selection strategy. We made it consistent in the whole section.**

***“We included journals that publish in the field of Medicine and in English…”***

***“Then, we filtered the journals by language (English) and subject (Medicine).”***

***Please note that some journals cover non-medical fields (in addition to the medical field) as shown in table 1.***

B. RCTs of what?

Again, some description will be needed. For example, does it include RCTs of public health interventions? Since health is conceived more broadly and therefore research also looks at wide ranging social determinants of health, there are also studies which look into these broader determinants?

From within the domain of biomedical sciences, there are wide-ranging sub-domains, say for example, the type of interventions - drugs, diagnostics, surgical interventions etc.

**Response: Thank you again. As clarified above, we included journals classified as having ‘medicine’ as their subject. Given our findings (null or very percentages for funding and COI being addressed) we believe that further classifying the studies will not be very informative.**

C. Characteristics of the journal: ?? clarity please?

**Response: Thanks for highlighting this. We edited that part of the ‘data abstraction’ section as follows:**

***“Characteristics of the journal (including impact factor, field of study, publisher)”***

-Discussion section:

D. It is important to present these findings against the backdrop of the journal policies on COI.

It is not adequately clear, if the lower number of COI declaration across the three concerned constituencies/stakeholders - authors, editors, and peer reviewers - is to do with the lack of journal policy making it mandatory; or it is the editors' lack of compliance with their own journal policy?

This an important aspect of this study. Suggest to include an explicit statement on this.

One of the major constraint of the manuscript is any reference to the journal policies of the journals from which the papers included in this COI review exercise/study. In absence of it, it is constrained to appreciate these results.

Again, it is not clear the basis of such a statement and any similar points in absence of any data presented regarding the policies of the journals which are included in this review study.

Comment on: “The percentage of articles with at least one author reporting presence of COI for our first sample was only 9%”. What might explain it? any conjectures?

Does it related to the way journals present 'guidelines for authors' or 'guidelines for peer reviewers etc.

In this manuscript, it is not clear as what have been the policies of journals on COI declarations. Seems it is important to view these findings against those policy brief of the respective journals.

If journals don't make COI declarations mandatory for all these concerned stakeholders - authors, editors, and reviewers - it a major gap.

**Response: Thank you for raising this important and key point. Whether the journals had policies to mandate COI declaration aross the three concerned groups (authors, editors, and peer revieweres) was not within the scope of our study. However, we have relied on previously published studies and added the following to the discussion section:**

***On the second hand, an article by Ralph et al. highlighted the fact that while there are concerns about authors’ financial COI in health research, there is little discussion on potential editorial financial COI [27]. In their sample of 20 public health journals, editor and peer reviewer financial COI policies were lacking strict disclosure requirements compared to author financial COI policies [27]. Moreover, another cross-sectional study of of high-impact medical specialty journals found that although 99% of those journals required author’s COI disclosure, only 12% required editorial’s COI disclosure [28]. A review of the COI policies of 72 health policy and services journals found that only one policy described how the COIs of the editorial team are managed during the editorial process [23].***

**We also added the following text to the discussion section:**

***One important question is whether the findings are explained by the lack of relevant journal policy or by the non-compliance of editors with such a policy. Reviews of the COI policies of journals in the clinical, surgical and public health areas did not address how the COIs of the editorial team are managed during the editorial process., raising the need for further exploration [29-31].***

E. But later the sub-section 4.3 presents some insights into how these findings compare with the existing scholarship in this area.

Some fine tuning of this point would be appropriate.

Elsewhere, authors said that this is first of its kind study.

? clarity? guidelines of their own journal?

**Response: Thanks again for highlighting this. To our knowledge and based on our literature review, our study is one of the first that examines the extent to which peer reviewers and editors consider study funding and authors’ COI, and the extent to which they report on their own COI. The article we referenced #25 highlighted the fact that while there is increased concerns on authors’ financial COI in health research, there is little discussion on potential editorial financial COI. In their sample of 20 public health journals, editor and peer reviewer financial conflict of interest policies were lacking strict disclosure requirements compared to author policies.**

**With regards to existing scholarship, and mentioned above, we have added the following text to part 4.3. of the discussion section:**

***“On the second hand, an article by Ralph et al. highlighted the fact that while there are concerns about authors’ financial COI in health research, there is little discussion on potential editorial financial COI [27]. In their sample of 20 public health journals, editor and peer reviewer financial COI policies were lacking strict disclosure requirements compared to author financial COI policies [27]. Moreover, another cross-sectional study of of high-impact medical specialty journals found that although 99% of those journals required author’s COI disclosure, only 12% required editorial’s COI disclosure [28]. A review of the COI policies of 72 health policy and services journals found that only one policy described how the COIs of the editorial team are managed during the editorial process [23].”***

**The above listed studies policies for handling COI in the peer review process, but none of them examines actual peer review reports.**

F. what relevance unpublished work has? Can be deleted.

“…published in non-open peer review journal.” This is a relevant point to make as part of limitation of the study.

**Response: Thanks for pointing this out. We considered this point carefully and prefer to keep the unpublished articles included in this statement. Our rational can be illustrated by the following example: According to our results, the majority of authors in the papers sampled from our selected journals came from high income countries (>60%) and only 5% were from low-income countries. These figures can be altered if rejected articles were to be considered. This is also in line with reviewer #1 comment: “Another interesting snippet was that the majority of authors in the papers sampled came from high income countries. Perhaps the acknowledged gap in analysis of papers rejected would tell us more”. For more clarify and precision, we have replaced ‘studies that were unpublished’ by ‘studies that did not survive the peer review process’.**

G. review? study?

Suggest to use same terms/terminology through the manuscript in the interest of clarity.

**Response: Thanks for highlighting this. We followed your suggestion. Please see below the edit:**

***“The results of our study provide insight…”***

-Tables:

H. Later in the Table 6, one of the parameters is "Authors (comments in their response letters)'. Does this mean that authors' rebuttals for some were available to access? And does this number (5 + 2 in Table 6) is of these 16 here in this Table?

As mentioned in my earlier comment, if only 16 instances of editors' letter being in the public domain, the denominator for this parameter need to be 16. Please clarify.

**Response: Thanks for your comment. Yes, authors' rebuttals were publicly accessible for all 144 studies included in the first sample, as these were deemed eligible specifically if they ‘publish all their peer review reports’ (open peer review). 5+2 is out of 144. And these were rebuttals to peer reviews and not specifically to editors’ comments. Independently, the same sample included 16 papers where there was public posting of editors’ letter to the authors, i.e., editors’ letters to the authors were not always posted publicly.**

2. It appears that a number of facets of COI reporting in relation to the three key constituencies - authors, reviewers, and editors - and presented in various tables in this manuscript need to be seen in the view of the editorial/journal policies and/or guidelines for authors /submission guidelines. I have tried to indicate such aspects and spots/places in the manuscript as indicative ones in the attached file (the manuscript with my review comments).

**Response: Thank you for this comment. We believe we have addressed this point in our response to comment 1 - D above.**

3. In this regard, it may mean to present some analysis of the journal policy/submission guidelines/guidelines for authors' of the journals included in this review study. This would help readers to know if many journal simply do not have policy documents that require authors, reviewers and editors to declare their COIs, or if it is to do with the implementation gap, that is, policies are in place but are not implemented with required diligence and rigor at the editorial offices. Suggestion to include such a section. If there might be constraints to present such an analysis, it certainly requires presentation of some insights from the existing scholarship in this area.

**Response: Thank you again for raising this point. As mentioned earlier, examining the policies of the journals is outside the scope of the study. But as suggested by the reviewer, we now reers to the existing scholarship in this area:**

***“On the second hand, an article by Ralph et al. highlighted the fact that while there are concerns about authors’ financial COI in health research, there is little discussion on potential editorial financial COI [27]. In their sample of 20 public health journals, editor and peer reviewer financial COI policies were lacking strict disclosure requirements compared to author financial COI policies [27]. Moreover, another cross-sectional study of of high-impact medical specialty journals found that although 99% of those journals required author’s COI disclosure, only 12% required editorial’s COI disclosure [28]. A review of the COI policies of 72 health policy and services journals found that only one policy described how the COIs of the editorial team are managed during the editorial process [23].”***

4. Under 'What is new?', it says, "This is one of the first studies that examines ... on their own COI." ( p no 4 and later in the submission). However, the basis of such a statement/claim is somewhat unclear as the section '4.3.Comparison to similar studies' compared study findings with some of the existing literature. A more fine tuned an nuance articulation would be more appropriate to keep it close to both the objectives of this review and more importantly findings of the review.

**Response: This point was addressed in number 1 question E. The studies listed under section 4.3 address policies for handling COI in the peer review process, but none of them examines actual peer review reports.**

5. It is not clear and not explained as why only two recent papers have selected from each of the journals included in the pool from where the papers are retrieved and included in this review. Please state it explicitly, unless I missed it. As well, it is not adequately as what parameters/criteria employed to determine which two recent papers to be included from many recent papers.

**Response: We selected 2 articles from each journal to achieve our desired sample size. Of the eligible articles, we include the two most recent based on their dates of publication (most recent).**

6. Similarly, the rationale for Sample 2, is somewhat weak on two counts: one, the authors might have already undertaken some work to decide on the scope of journals/type of articles to be included in this review for COIs etc; two, it is said that a paper (ref no 25 in the manuscript) led the authors to include RCTs as Sample 2. Both appear to be somewhat ad hoc rationales, especially the latter. Will be helpful and will be needed to present more robust rationale for sample 2. Readers would expect a rationale that is more informed and well-thought which might be the case. However, the current articulation seems like it was 'by chance'.

**Response: Thank you for this comment. The selection of the 2^nd^ sample was indeed ad hoc and in order to achieve meaningful conclusions as explained in the methods section:**

***“First, we included a sample with any type of original research. However, we found that a very low percentage (9%) had at least one author reporting presence of COI. Given this would not allow us to achieve the study objective, we decided to collect a second sample restricted to randomized controlled trials (RCTs) as a survey had found that more than half of clinical trials had authors reporting presence of COI [24].”***

7. Please you may ignore highlighters in the attached file with my embedded review comments.

**Response: Noted, thank you.**
